# Regulated expression of matrix metalloproteinases, inflammatory mediators, and endometrial matrix remodeling by 17beta-estradiol in the immature rat uterus

**DOI:** 10.1186/1477-7827-7-124

**Published:** 2009-11-04

**Authors:** Louise A Russo, Bryan J Peano, Shreya P Trivedi, Todd D Cavalcanto, Benjamin A Olenchock, Joseph A Caruso, Amanda R Smolock, Oleg Vishnevsky, Russell M Gardner

**Affiliations:** 1 Villanova University Department of Biology, 800 Lancaster Avenue, Villanova, PA 19085, USA

## Abstract

**Background:**

Administration of a single physiological dose of 17beta-estradiol (E2:40 microg/kg) to the ovariectomized immature rat rapidly induces uterine growth and remodeling. The response is characterized by changes in endometrial stromal architecture during an inflammatory-like response that likely involves activated matrix-metalloproteinases (MMPs). While estrogen is known as an inducer of endometrial growth, its role in specific expression of MMP family members in vivo is poorly characterized. E2-induced changes in MMP-2, -3, -7, and -9 mRNA and protein expression were analyzed to survey regulation along an extended time course 0-72 hours post-treatment. Because E2 effects inflammatory-like changes that may alter MMP expression, we assessed changes in tissue levels of TNF-alpha and MCP-1, and we utilized dexamethasone (600 microg/kg) to better understand the role of inflammation on matrix remodeling.

**Methods:**

Ovariectomized 21 day-old female Sprague-Dawley rats were administered E2 and uterine tissues were extracted and prepared for transmission electron microscopy (TEM), mRNA extraction and real-time RT-PCR, protein extraction and Western blot, or gelatin zymography. In inhibitor studies, pretreatment compounds were administered prior to E2 and tissues were harvested at 4 hours post-hormone challenge.

**Results:**

Using a novel TEM method to quantitatively assess changes in stromal collagen density, we show that E2-induced matrix remodeling is rapid in onset (< 1 hour) and leads to a 70% reduction in collagen density by 4 hours. Matrix remodeling is MMP-dependent, as pretreatment with batimastat ablates the hormone effect. MMP-3, -7, and -9 and inflammatory markers (TNF-alpha and MCP-1) are transiently upregulated with peak expression at 4 hours post-E2 treatment. MMP-2 expression is increased by E2 but highest expression and activity occur later in the response (48 hours). Dexamethasone inhibits E2-modulated changes in collagen density and expression of MMPs although these effects are variable. Dexamethasone upregulates MMP-3 mRNA but not protein levels, inhibiting E2-induced upregulation of MMP-7, and -9, and MCP-1 mRNA and protein but not inhibiting the hormone-induced increase in TNF-alpha mRNA.

**Conclusion:**

The data demonstrate that E2-regulated endometrial remodeling is rapid in onset (<1 hour) and peak expression of MMPs and inflammatory mediators correlates temporally with the period of lowest stromal collagen density during uterine tissue hypertrophy.

## Background

The ovariectomized (OVX) immature rat has been used extensively to study hormone-specific regulatory processes in the mammalian uterus. In this model, administration of a single physiological dose of 17β-estradiol (E_2_; 40 μg/kg) induces a biphasic uterine growth response, characterized by rapid tissue hypertrophy followed by hyperplasia. The early hypertrophic growth phase peaks approximately 4 h after E_2 _administration and is characterized by extensive collagen matrix remodeling of the endometrial stroma [1]. The controlled degradation and repair of the stromal extracellular matrix is a necessary component of a variety of normal reproductive processes, including endometrial cycling [2-4], implantation [5], and pregnancy [6], and involves regulated expression and activity of proteolytic enzymes including the matrix metalloproteinases (MMPs) by steroid hormones. Hormone-induced regulation of MMP expression is complex and appears to involve cell-type specific changes in expression of local paracrine mediators as well as direct and indirect effects on mRNA transcription via activated receptors.

Matrix metalloproteinases comprise a family of related enzymes, each of which can degrade at least one component of the extracellular matrix, consisting of numerous types of collagen, gelatin, elastin, fibronectin, and laminin, among others [reviewed in [7]. In addition, MMPs have been shown to act on non-matrix substrates by modifying and/or activating other MMPs [8-10], growth factors [11], cytokines [12,13], chemokines [14,15], and other growth regulators that are matrix-bound or present on the cell surface [16,17]. Expression is controlled at the level of transcription in response to proto-oncogenes and exogenous signals, including hormones [18,19], cytokines [20,21], and growth factors [22]. At the post-translational level, MMPs in the extracellular space are regulated by endogenous inhibitors, including a group of tissue-derived inhibitors termed TIMPs (tissue inhibitors of metalloproteinases) [7,23]. Furthermore, most MMPs are secreted as inactive proenzymes (proMMPs), also known as zymogens, requiring the cleavage of a specific propeptide domain for activation [24]. Although the mechanisms governing proMMP activation are not completely understood, evidence suggests that inflammatory mediators function to regulate MMP activity in the extracellular space [4,9,20,25].

Many studies have shown that administration of physiological dose level of estrogen to the immature or OVX rodent induces an acute inflammatory-like response, including the coordinated influx of blood-born macrophages, neutrophils, and eosinophils into the uterine endometrial stroma [26-31]. The pro-inflammatory and growth effects of E_2 _are antagonized by co-administration of anti-inflammatory agents, including the synthetic glucocorticoid dexamethasone [32-36] and the steroid hormone progesterone (P_4_) [37]. Evidence suggests that the estrogen-mediated infiltration of inflammatory cells into the endometrial stroma may lead to activation of extracellular proMMPs, which have been shown to colocalize with leukocytes in the mouse [38] and human [29] endometrium. Proteinases released by inflammatory cells, including tryptase, chymase, leukocyte elastase, and MMPs, have been shown to activate proMMPs in culture [8,9,39,40], while metabolites and oxidants generated by inflammatory cells may also regulate MMP activation [41-43]. These studies and others suggest an important role for inflammatory mediators in the activation of proMMPs in the endometrial extracellular matrix. In turn, activated MMPs have been shown to modulate inflammatory processes, including the activity and mobilization of chemokines and cytokines [44,45] thereby regulating the further influx of inflammatory cells [11-16]. Therefore, MMPs and inflammatory mediators are components of a complex network governing uterine growth and remodeling, as well as other normal and disease processes.

In the current study, we have developed a novel method to quantitatively analyze transmission electron micrographs to assess estrogen-induced endometrial collagen remodeling in the OVX immature rat model along a time course that includes both the hypertrophic and hyperplastic phases of uterine growth. Using matrix collagen density as a quantitative and reliable endpoint, we specifically determined that estrogen-induced stromal matrix remodeling is MMP-dependent. We further characterized hormone-regulated expression of MMP-3 (stromelysin-1), MMP-7 (matrilysin), MMP-2 (gelatinase A), and MMP-9 (gelatinase B), four metalloproteinases believed to function prominently in various reproductive processes [6,38], as well as the inflammatory mediators TNF-α and MCP-1 along an extended post E_2_-treatment time course to assess changes in tissue protease and inflammatory potential through both the early hypertrophic and later hyperplastic growth phases. In addition, we have investigated the effect of the anti-inflammatory synthetic glucocorticoid dexamethasone on E_2_-induced stromal matrix remodeling and MMP and inflammatory mediator expression to better understand the role of inflammation in the tissue pathway linking estrogen receptor activation to changes in tissue ultrastructure necessary for uterine growth and remodeling.

## Methods

### Animal treatments and tissue preparations

All animal experiments were performed in compliance with NIH standards as specified in the *Guide for Care and Use of Laboratory Animals *(copyright 1996, National Academy of Sciences). Female Sprague-Dawley rats (Ace Animal Corp., Boyertown, PA) were ovariectomized at 21 days of age and allowed to recover for 3-5 days. For E_2 _time course experiments, animals were administered a single physiological dose of E_2 _(40 μg/kg in a 0.9% NaCl, 0.4% EtOH vehicle) by intraperitoneal (i.p.) injection at the indicated time intervals prior to tissue collection at necropsy. This in vivo dose level of E_2 _has been shown to induce changes in uterine wet weight, tissue architecture, and gene expression characteristic of estrogen receptor activation [1,46-48]. For all other experiments, animals were i.p. administered a single 40 μg/kg bolus of E_2 _4 h prior to tissue harvest, while control animals received vehicle only in all studies. Batimastat was administered i.p. at a dose level (BB-9; 40 mg/kg in a 1× PBS, 0.1% Tween-20 vehicle) shown to be effective at inhibiting MMPs in vivo [49] 4 h prior to E_2 _or saline control. Dexamethasone was administered i.p. at a dose level of 600 μg/kg in a 0.9% NaCl, 10% EtOH vehicle that has been previously used in rodent studies to assess in vivo steroid specific responses in the uterus [46-48]. Animals were treated twice with dexamethasone (600 μg/kg) at 20 and 4 h before E_2 _or saline control administration to elicit both early and late gene effects of the activated glucocorticoid receptor (GR) that may affect uterine responsiveness to E_2 _via genomic or nongenomic mechanisms. Batimastat was generously provided by GlaxoSmithKline, while other treatment compounds were obtained from Sigma-Aldrich (USA). Dose levels of all compounds were administered at injection volumes of 1 ml/100 g body weight. Inhibitor compounds were administered i.p., with control animals receiving the corresponding vehicle solution i.p. at the same treatment times as inhibitor injections.

For uterine tissue collection at necropsy, animals were euthanized by decapitation on the same day in accordance with the specific treatment time interval and the tissue was stripped of fat and mesentery, weighed, and the horns separated. One horn from each animal was flash frozen in liquid N_2 _and stored at -80°C for later RNA purification. For protein analyses, two isolated horns from different animals were pooled and fresh-processed for total protein extraction. For transmission electron microscopy analyses, tissues were immediately fixed in 2.5% glutaraldehyde in 0.1 M phosphate buffer.

### Transmission electron microscopy (TEM)

Individual uteri were fixed in 2.5% glutaraldehyde in 0.1 M phosphate buffer, pH 7.4, and 2 mm-cross sections of each uterine horn were cut and incubated overnight at 4°C in 2.5% glutaraldehyde in 0.1 M phosphate buffer. Glutaraldehyde buffer was removed, and the samples were rinsed three times (15' each) in 0.1 M phosphate buffer. Samples were incubated in 1% osmium in 0.1 M phosphate buffer, rinsed, and dehydrated in a series of ethanol washes. Samples were incubated twice for 5' in propylene oxide and then transferred to a rotor for 1 h at room temperature in a 1:1 mixture of propylene oxide and epon [47% Embed 812, 31% DDSA (dodenyl succinic anhydride), 19% NMA (nadic methyl anhydride), 3% BDMA (benzyldimethylamine)], followed by an overnight incubation in 1:2 propylene oxide-epon, and finally 100% epon for 2-3 hrs. Individual uterine samples were embedded in 100% epon in silicon flat embedding molds, and capsules were polymerized in a 60°C oven for over 48 hrs. Ultrathin transverse sections (70 nm) were prepared using a diamond knife (Diatome) on a MT 6000-XL ultramicrotome, captured on 300-mesh copper grids, and stained with 2% uranyl acetate. All reagents and materials were obtained from Electron Microscopy Sciences (Hatfield, PA).

A minimum of 3 blocks with tissue from different areas along the length of each uterine horn were chosen at random from a total pool of 5-6 blocks and were used to prepare ultrathin sections to provide a comprehensive survey of whole organ ultrastructure representatively. A minimum of 6 transverse sections were prepared per tissue block and viewed using a Hitachi 7600 microscope fitted with an AMT digital camera. Visual analyses and micrograph surveys were restricted to the endometrial stromal compartment at a minimum of 3 random sites within the endometrium of each section so as to avoid bias. At each random site within the endometrial stromal matrix compartment, 3-6 digital photomicrographs at 12,000× magnification were taken. For quantitative analysis of collagen density, the full area of photomicrographs was digitally fitted with an 8 × 8 square grid containing a total of 64 cross-points, and each cross-point was evaluated to determine the structure that it overlaid. Image J software (NIH) was used to quantify the total number of crossbars landing on collagen fibrils (positive hits), and the values were expressed as a ratio to the total number of crossbars to determine a percent matrix density volume for each photomicrograph. Stromal areas with a high percentage of collagen fibrils cut in transverse were selected to best ensure that quantitative crossbar analysis was not significantly affected by dramatic differences in collagen fiber orientation. A total of 36-75 micrographs were analyzed per time point or treatment condition. Values for all micrographs for each animal were averaged, and statistical differences between averages of each time point or treatment group were analyzed using one-way ANOVA. Error bars on graphs represent standard error, and a standard P value of ≤ 0.05 was considered significant.

### RNA isolation and quantitative real-time PCR

For TaqMan^® ^quantitative real-time PCR analyses of mRNA levels, total RNA was prepared individually from the uterus of each rat. Each uterus was disrupted for 2-3 minutes in 0.75 mL QIAzol lysis reagent (Qiagen, Valencia, CA) using a 5 mm bead in a TissueLyser (Qiagen-Retsch). The homogenate was subjected to a 0.2 mL chloroform extraction and centrifugation at 4°C for 15 min at 20,000 × *g*, and 0.35 mL aqueous phase was collected for RNA purification using an RNeasy kit (Qiagen) according to the manufacturer's protocol. Residual genomic DNA was removed by on-column RNase-free DNase treatment during RNA purification, and the RNA concentration was adjusted to 0.05 mg/mL. Messenger RNA levels were analyzed using quantitative real-time PCR on an ABI Prism 7900 Sequence Detection System and TaqMan^® ^gene expression assays (Applied Biosystems, Foster City, CA) for MMP-2 (Rn01538174_m1), MMP-3 (Rn00591740_m1), MMP-7 (Rn00563467_m1), MMP-9 (Rn00579162_m1), TNF-α (Rn01753871_m1), MCP-1 (Rn01456716_g1) and eukaryotic 18s rRNA (Hs99999901_s1). To compare mRNA expression levels among samples, mRNA for each gene of interest was normalized to the expression of a housekeeping gene, 18s rRNA, and differences in gene expression were calculated as a fold change to the mean of the vehicle group.

### Protein extraction, SDS-PAGE, and immunoblotting

Detailed methods for protein extraction, SDS-PAGE, and immunoblotting have been described previously [46]. In brief, 2-3 uterine tissues were pooled and homogenized in 50 mM Tris pH 7.5, 150 mM NaCl, 1 mM CaCl_2_, 0.05% Brij 35, 1 mM PMSF, and 10 μg/ml leupeptin using a polytron homogenizer (Brinkman Instruments, Westbury, NY). Homogenates were centrifuged at 15,000 × g for 30 minutes at 4°C and the supernatants removed and protein quantified. Extracted total protein (20 μg) samples were loaded into the wells of a pre-cast gel (4-20% Tris-HCl; BioRad, Hercules, CA) and resolved by electrophoresis in a Mini-Protein II module (BioRad) according to the manufacturer's protocol. Resolved proteins were transferred to an Immobilon-P membrane (Millipore, Billerica, MA), which was blocked (5% BSA in 1× Tris-buffered saline + 0.1% Tween-20 + 2% normal goat serum) and then incubated in a 1:2000 dilution of affinity-purified polyclonal rabbit anti-MMP-2 IgG (Novus Biologicals, Littleton, CO; #NB200-193), anti-human MMP-7 IgG (Lifespan Bioscience, Seattle, WA; #LS-B1237), anti-MMP-3 IgG (Abcam, Cambridge, MA; #ab53015), anti-MMP-9 IgG (Abcam #ab7299), anti-TNF-α IgG (Cedarlane, Burlington, NC; #CL9572AP), anti-MCP-1 IgG (Millipore, Billerica, MA; #AB1834P) or monoclonal mouse anti-β actin IgG (Abcam #ab6276) overnight in blocking solution at 4°C with gentle agitation. Membranes were washed and incubated in a 1:10,000 dilution of secondary HRP-conjugated goat α-rabbit IgG or α-mouse IgG (Jackson Immunologicals, West Grove, PA) in blocking solution and then washed and transferred to a solution of SuperSignal Substrate reagents (Pierce/Thermo Scientific, Rockford, IL) for enhanced chemiluminescent signal detection, imaged, and analyzed using the FluorChem SA system (Alpha Innotech, San Leandro, CA) and AlphaEase FC software. Band intensities were measured as integrated density volumes (IDV) and expressed as % of control lane values for comparisons between treatment groups. Specificity of bands identified using primary antibodies was confirmed by exposing sample blots to either 2% normal goat serum alone, secondary goat HRP-conjugated antibody in the absence of primary antibody, or normal rabbit IgG (Jackson Immunologicals) only prior to secondary antibody incubation.

### Gelatin zymography

Extracted total protein (20 μg) samples were mixed 1:1 with zymography sample buffer (Bio-Rad) and incubated at room temperature for 10 min. Samples were loaded into the wells of a pre-cast gel (10% gelatin, BioRad) and resolved by electrophoresis in a Mini-Protein II module (BioRad) under non-reducing conditions. Active human MMP-2 (Calbiochem/EMD Biosciences, Gibbstown, NJ; #PF023) was loaded in one lane of the gel as a control. After electrophoretic separation, the gel was washed twice for 30 minutes each in 1× renaturation buffer (Bio-Rad) at room temperature (RT) with gentle agitation and then incubated in 1× development buffer (Bio-Rad) for 30 minutes at RT. Fresh development buffer was added and the gel incubated at 37°C for 18-24 hours. Developed gels were stained with Coomassie Brilliant Blue R-250 (0.5% in 30% methanol, 10% acetic acid) for 30 minutes at RT. The gel was destained in 30% methanol, 10% acetic acid until clear bands representing proteolytic areas were visualized. Gels were visualized with the FluorChem SA system (Alpha Innotech) and analyzed using AlphaEase FC software. Band intensities were measured as integrated density volumes (IDV) and expressed as % of control lane values for comparisons between treatment groups.

### Statistics

Data is represented as the average ± standard error of the mean (SE). Data were analyzed initially via one-way ANOVA followed by Tukey's Honestly Significant post-comparison test to assess differences between treatment groups. P values ≤ 0.05 were considered significantly different.

## Results

### E_2 _induces rapid remodeling of uterine stromal matrix ultrastructure

**Figure 1:**
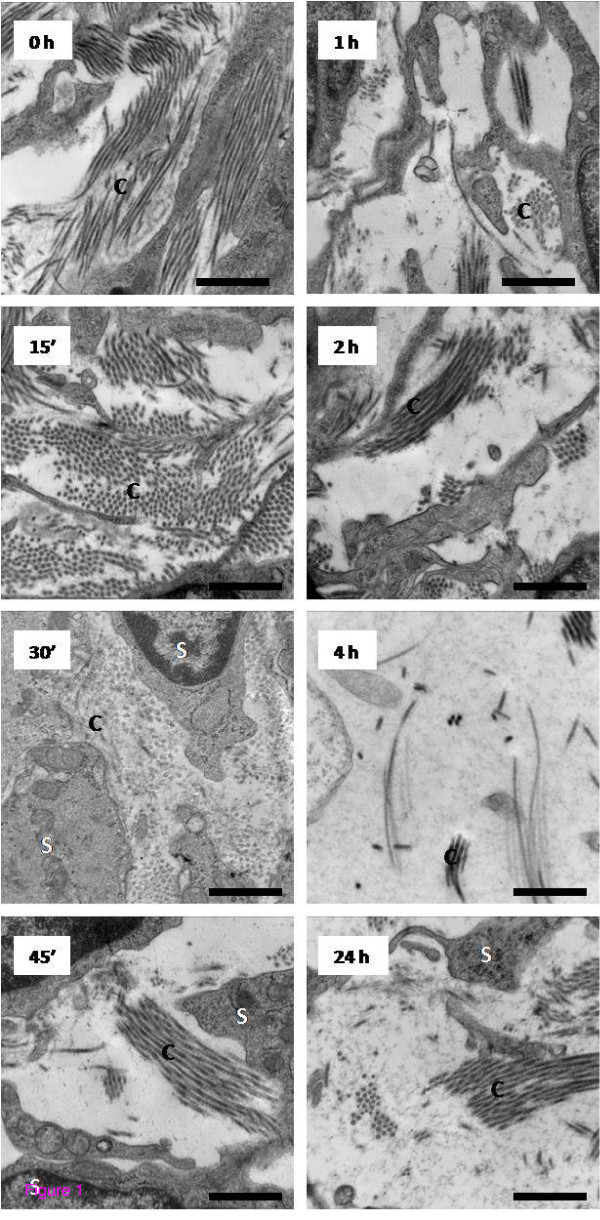
**Transmission electron microscopy analysis of ultrastructural changes in the uterine stromal matrix in response to E_2 _treatment**. Representative TEM micrographs of the endometrial stroma of animals administered E_2 _(40 μg/kg) at the indicated time points prior to tissue collection. Micrographs shown are representative of typical observations from a total of 54 to 72 micrographs analyzed from 4-5 animals per time point; C = Collagen, S = Stromal cell, scale bar = 0.5 μm.

**Figure 2:**
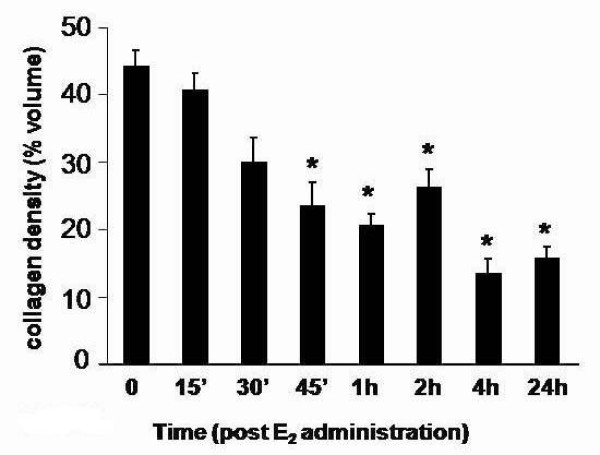
**Quantitative analysis of E_2_-induced changes in uterine stromal matrix density**. Endometrial collagen density values represent mean percent volume per time point calculated from micrograph analysis; one-way ANOVA, error bars represent ± SE for 4-5 animals per time point, total micrographs analyzed per time point range from 54 to 72; *P < 0.05 compared to 0 h saline control.

To better characterize previously described E_2_-induced changes in uterine stromal collagen matrix remodeling [1], we developed a novel method to quantitatively assess changes in collagen fibril density using a grid overlay analysis of TEM photomicrographs (as described in Methods). This method establishes matrix density as a quantifiable endpoint for studies aimed at assessing changes in activity of matrix processing enzymes, including the MMPs, induced by bioactive compounds and inhibitors in a very precise and direct manner. We assessed collagen matrix density using micrographs prepared from uterine sections along the entire length of the uterine horn to best represent whole tissue architecture changes and over an extended time course after administration of a single physiological dose of E_2 _(40 μ/kg) to study stromal collagen architecture throughout both the early hypertrophic and latter hyperplastic phases of uterine growth. Qualitative assessment of micrographs indicates that a dense irregular organization of collagen fibers is clearly observed within saline-treated tissues (0 h), but this organization rapidly disappears such that a significant difference is evident within 45 minutes of hormone administration. Matrix density reaches a low point by 4 h when only a few isolated fibrils within the stromal compartment remain (Figure 1). Quantitative analysis of collagen matrix density supports a significant decrease (~30% less than 0 h-saline levels) within 45 minutes post-E_2 _treatment and a continual decline over time, reaching its lowest value (~70% less than 0 hr) at 4 h and remaining low through the 24 h treatment interval (Figure 2).

### Stromal matrix remodeling is dependent upon activity of MMPs

**Figure 3:**
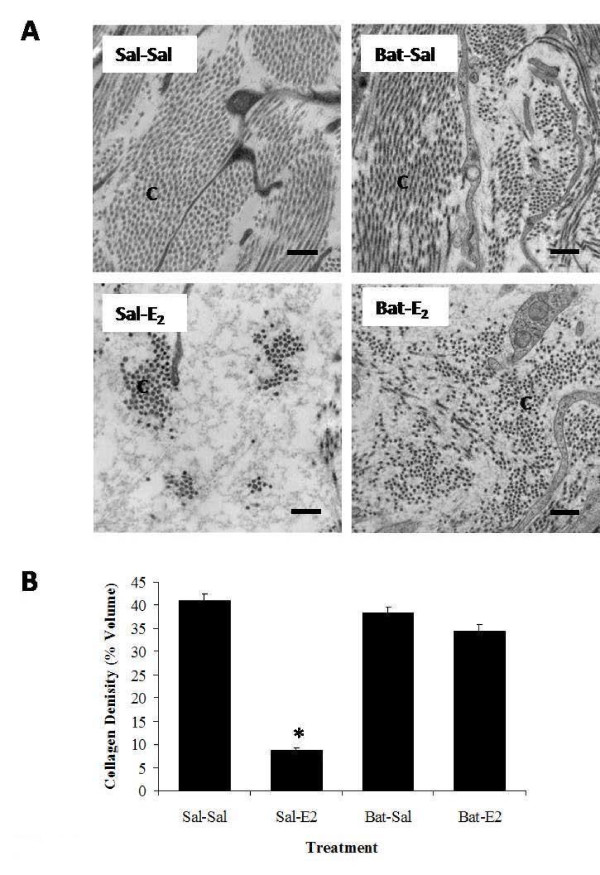
**Batimastat effects on E_2_-induced changes in uterine stromal matrix density**. Transmission electron microscopy analysis of endometrial stroma from animals pretreated with saline or batimastat (Bat: 40 mg/kg) 4 h prior to administration of E_2 _or saline injected 4 h prior to tissue collection. Panel A: Representative TEM micrographs of stromal matrix. Micrographs shown are typical from a total of 36 to 54 micrographs analyzed from 3-4 animals per treatment group; C = Collagen, S = Stromal cell, scale bar = 0.5 μm. Panel B: Quantified endometrial collagen density; one-way ANOVA, error bars represent ± SE for 3-4 animals per treatment group, total micrographs analyzed per time point range from 36 to 54; *P < 0.05 compared to saline control (Sal-Sal).

Upon establishing a quantitative method to assess changes in stromal matrix density, we assessed effects of a broad-range hydroxamate inhibitor of MMPs, batimastat (40 mg/kg), on E_2_-induced matrix remodeling in OVX animals (Figure 3) to better characterize the role of activated MMPs in hormone-stimulated collagen reorganization. Matrix density was analyzed in saline- or batimastat-pretreated animals 4 h after E_2 _administration, the time point at which collagen density was observed to be at its lowest after hormone treatment (Figures 1 and 2). Transmission electron microscopy assessment (Figure 3) shows that batimastat alone has no effect on the stromal collagen network (Sal-Sal vs. Bat-Sal), but overall collagen density in batimastat-pretreated E_2_-induced animals (Bat-E_2_) is significantly higher (~4-fold) compared to those receiving saline pretreatment and E_2 _treatment (Sal-E_2_), clearly indicating that E_2_-induced uterine matrix remodeling is, at least in part, dependent upon activated MMPs.

### E_2 _induces transient upregulation of uterine MMP mRNA and protein

**Figure 4:**
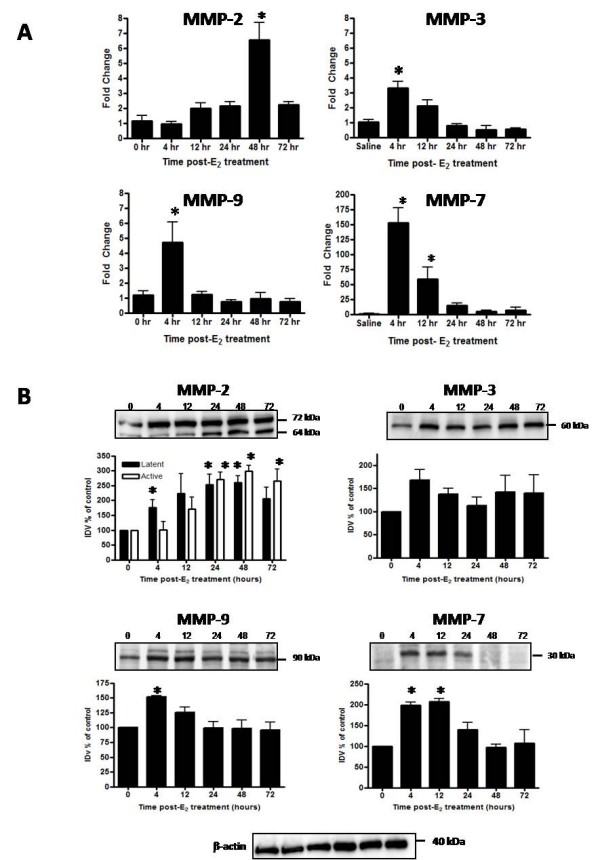
**Temporal changes in uterine MMP mRNA and protein in response to E_2_**. Uterine mRNA analysis via TaqMan^® ^real-time PCR (panel A) and protein analysis via Western blot (panel B) are shown for MMP-2, MMP-3, MMP-7 and MMP-9 from uteri of OVX rats 0-72 h after treatment with saline (0 h control) or E_2 _(40 μg/kg). Messenger RNA values (panel A) represent mean ± SE, n = 6 tissues per group; one way ANOVA, *P < 0.05 compared to 0 h time point. Western blot images (panel B) are representative of a minimum of 3 blots using 3 separate pools of total protein extracted from different animals. Intensities of protein bands were quantitated using AlphaEase FC software (Alpha Innotech) as described in the Methods and values for each time point expressed as percent of saline control (100%). Quantitative data (panel B) reflect average band intensity values ± SE measured from ≥ 3 blots with total protein extracted from different animals in 2 separate experiments; one way ANOVA, *P < 0.05 compared to 0 h time point. IDV = integrated density value. Western blot analysis of β-actin as a control for gel loading is shown for the protein samples used to assess MMP protein for the blots above.

While effects of ovarian hormones on uterine MMP expression have been studied under a variety of conditions in the rodent and human, we chose to fully characterize temporal changes in expression of MMP-2, MMP-3, MMP-7, and MMP-9 induced specifically by E_2 _in the immature OVX rat and to examine any correlation in enzyme levels with the time course of collagen matrix remodeling. The enzymes surveyed include those known to be expressed by uterine epithelial (MMP-7) as well as stromal (MMP-2, MMP-3 and MMP-9) cells [50]. In addition, collagen substrates for MMP-2 (I, III, IV, V, VII, X, XI), MMP-3 (II, III, IV, V, VII, IX, X), MMP-7 (I, IV, X), and MMP-9 (IV, V, VII, XIV) have been characterized in vitro [50]. Levels of mRNA and protein for these MMPs were assessed following saline treatment (0 h) and at 5 intervals (4-72 h) post-E_2 _administration. While mRNA transcripts for all MMPs analyzed (Figure 4A) are detected in the uterus in saline control tissues (0 h), significant increases in transcript levels are induced within 4 h by E_2 _treatment for MMP-3, MMP-7, and MMP-9. Of these enzymes, the fold induction (~150-fold) for MMP-7 is clearly the most robust (Figure 4). In contrast, levels of MMP-2 mRNA do not increase until later in the time course, with peak fold induction (~7 fold) occurring at 48 h post-hormone administration, thereby placing it on a different time line of regulation compared to MMP-3, MMP-7, and MMP-9. Upregulation of mRNA for all enzymes is transient, as transcript levels decline significantly and return to control values by 72 h.

Western blot analyses (Figure 4B) were completed to determine if uterine MMP protein changes in correlation with the mRNA induction profiles for each enzyme. MMP-2, MMP-3, and MMP-9 are detected in saline tissues and increase after E_2 _treatment in accord with peak changes in mRNA for each gene. Levels of the latent (~72 kDa) form of MMP-2 increase significantly within 4 h, but peak changes in latent and active (~64 kDa) protein do not occur until ~24-48 h post-E_2 _treatment (Figure 4B), which correlates temporally with the peak in mRNA content (Figure 4A). Tissue levels of MMP-3 (~62 kDa) and MMP-9 (~90 kDa) increase within 4 h of E_2 _treatment (Figure 4B) in conjunction with changes in mRNA (Figure 4A). While levels of MMP-9 decline over time, reaching control values within 24 h, MMP-3 protein remains elevated through 72 h, although the mRNA for this enzyme reaches control levels within 24 h after hormone administration (Figure 4A). This indicates that MMP-3 protein is temporally stabilized and levels do not decline to those observed prior to E_2 _stimulation. Latent MMP-7 (~29 kDa) is not detectable in control tissues (0 h), but levels of the enzyme increase significantly by 4 h post-E_2 _treatment, then decline over time to return to control values by 48 h (Figure 4B) in correlation to the temporal pattern of mRNA expression (Figure 4A). Upregulation of MMP-3, MMP-7, and MMP-9 protein expression 4 h post-E_2 _indicates that these enzymes are in fact elevated rapidly by hormone treatment with peak increases in protein content occurring at the time point when stromal collagen matrix is lowest (Figures 1 and 2).

**Figure 5:**
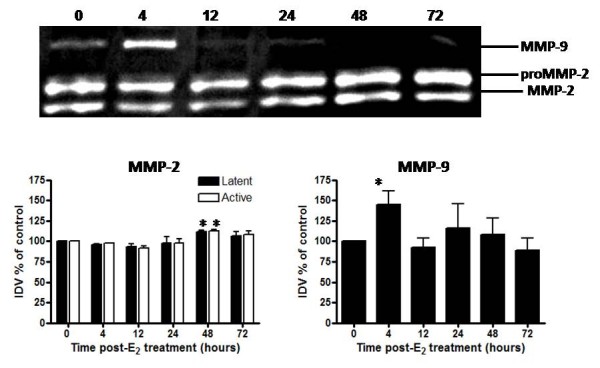
**Temporal changes in gelatinase activity in response to E_2_**. Gelatin zymography analysis is shown for MMP-2 and MMP-9 activity from uteri of OVX rats 0-72 h after treatment with saline (0 h control) or E_2 _(40 μg/kg). Zymogram (top) is representative of 6 gels using 3 separate pools of total protein extracted from different animals. Quantitative data (bottom) represents analysis of band intensity using AlphaEase FC software (Alpha Innotech) as described in the Methods; one way ANOVA, values represent mean ± SE for 6 gels. *P < 0.05 compared to 0 h time point.

E_2_-induced changes in activity of the gelatinase enzymes were assessed via gelatin zymography (Figure 5) to confirm overall protein expression levels observed via Western blot. The major bands representing gelatin-degrading activity were identified as active MMP-9 (~90 kDa) and latent and active MMP-2 (~72 kDa and 64 kDa, respectively). Overall activity of MMP-2 was greater than that of MMP-9 based upon band intensities. Levels of active MMP-2 decrease slightly within 4-12 h of hormone treatment (Figure 5). However, both latent and active forms of MMP-2 increase by 48 h post-E_2 _treatment, with peak activity correlating temporally with the highest level of total protein observed via Western blot (Figure 4). Activity of MMP-9 increases significantly at 4 h post E_2_-treatment (Figure 5) also corresponding to the time point when total protein levels are highest (Figure 4). Activity of MMP-3 and MMP-7 was assessed via casein gel zymography, however sensitivity of the assay was not sufficient to permit quantitative analysis (data not shown).

### E_2 _induces transient upregulation of the pro-inflammatory cytokine TNF-α and the β-chemokine MCP-1

**Figure 6:**
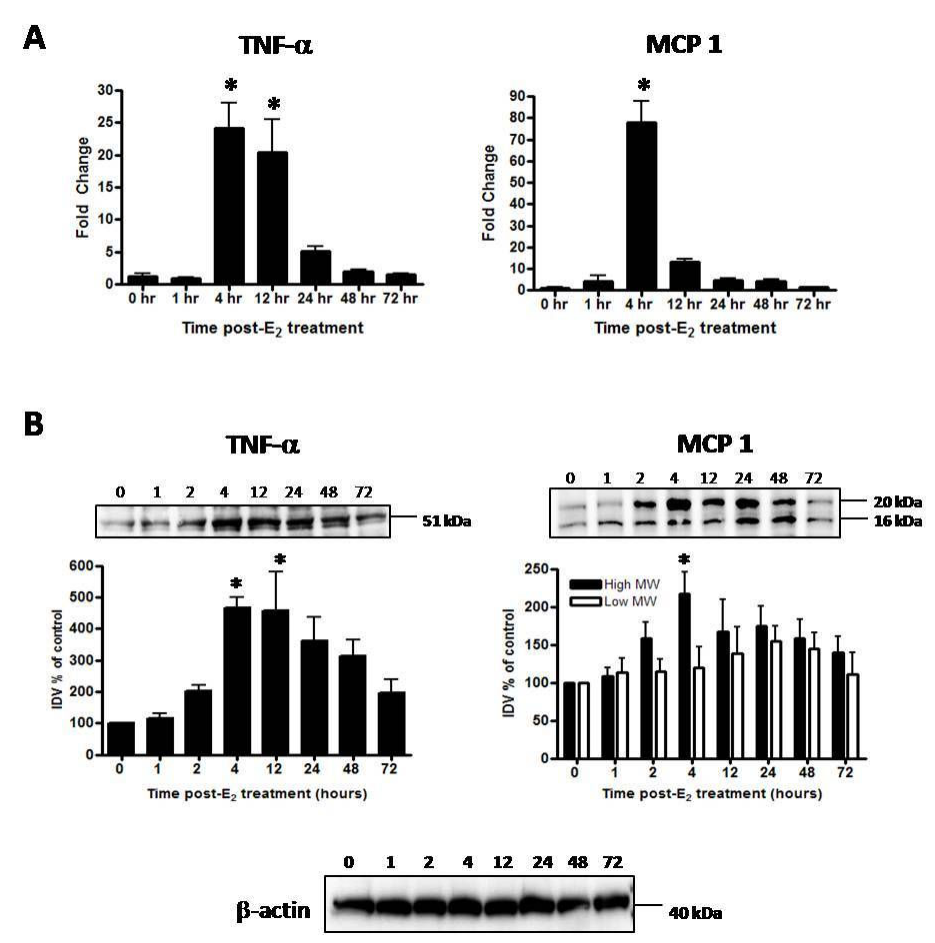
**Temporal changes in uterine cytokine mRNA and protein in response to E_2_**. Uterine mRNA analysis via TaqMan^® ^real-time PCR (panel A) and protein analysis via Western blot (panel B) are shown for TNF-α and MCP-1 from uteri of OVX rats 0-72 h after treatment with saline (0 h control) or E_2 _(40 μg/kg). Messenger RNA values (panel A) represent mean ± SE, n = 5-6 per group; one way ANOVA, *P < 0.05 compared to 0 h time point. Western blots images shown (panel B) are representative of a minimum of 3 blots using 3 separate pools of total protein extracted from different animals in 2 separate experiments. Intensities of protein bands were quantitated using AlphaEase FC software (Alpha Innotech) as described in the Methods and values for each time point expressed as percent of saline control (100%). Quantitative data (panel B) reflect average band intensity values ± SE measured from ≥ 3 blots with total protein extracted from different animals in 2 separate experiments; one way ANOVA, *P < 0.05 compared to 0 h time point. IDV = integrated density value. Western blot analysis of β-actin as a control for gel loading is shown for the protein samples used to assess TNF-α and MCP-1 protein for the blots above.

E_2_-induced changes in expression of the pro-inflammatory cytokine, TNF-α, and the β-chemokine MCP-1, were assessed over the biphasic growth response to better characterize the tissue inflammatory response and to compare changes temporally with matrix processing and MMP expression. Levels of TNF-α mRNA and protein transiently increase with a peak in expression occurring 4 h after hormone treatment (Figure 6A). TNF-α mRNA increases nearly 25 fold at 4 h and remains elevated through 12 h post-E_2 _treatment before declining to control values by 48 h. Western blot analysis (Figure 6B) identified the homotrimeric form of the protein (~51 kDa) as well as lower MW forms. Analysis of the receptor-ligand homotrimeric form shows slight elevation of protein early (1-2 h) with maximum levels occurring at 4 and 12 h post E_2 _administration (Figure 6B), correlating temporally to the major time points of mRNA elevation. This pattern clearly indicates a potent overall enhancement of tissue levels of TNF-α expression, most notably during the early hypertrophic period of uterine growth following hormone treatment. MCP-1 mRNA expression is also significantly upregulated (~80 fold) 4 h post-E_2 _treatment. While mRNA levels return to control values within 24 h, MCP-1 protein increases by 4 h but remains elevated through 48 h before returning to control tissue levels 72 h post-treatment (Figure 6B). Western blot analysis detects a higher MW form of MCP-1 (~20 kDa) which may represent an intact bioactive form of the chemokine, as well as a lower MW form (~16 kDa) which may represent a proteolytic and receptor-inactive product (Figure 6B). Blot analysis indicates that levels of the higher MW MCP-1 product peak at 4 h whereas the lower MW product peaks at ~24 h post treatment. E_2 _treatment thus rapidly increases levels of two potent pro-inflammatory chemicals, TNF-α and MCP-1, and the induction correlates temporally with the hypertrophic phase of tissue growth, which is characterized by tissue edema and stromal matrix remodeling.

### Dexamethasone pretreatment inhibits E_2_-induced uterine growth and changes in uterine tissue ultrastructure

**Figure 7:**
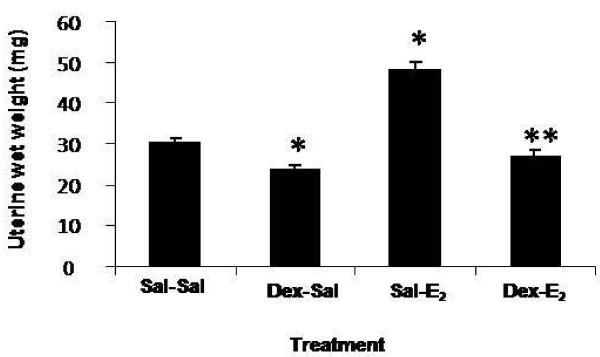
**Dexamethasone effects on uterine wet weights**. Animals were pretreated with dexamethasone (Dex: 600 μg/kg) or saline 20 h and 4 h prior to administration of E_2 _or saline injected 4 h before tissue collection. Values represent mean ± SE for 12-14 animals per treatment group; one way ANOVA, * P < 0.05 compared to saline control (Sal-Sal), ** P < 0.05 compared to E_2 _control (Sal-E_2_).

**Figure 8:**
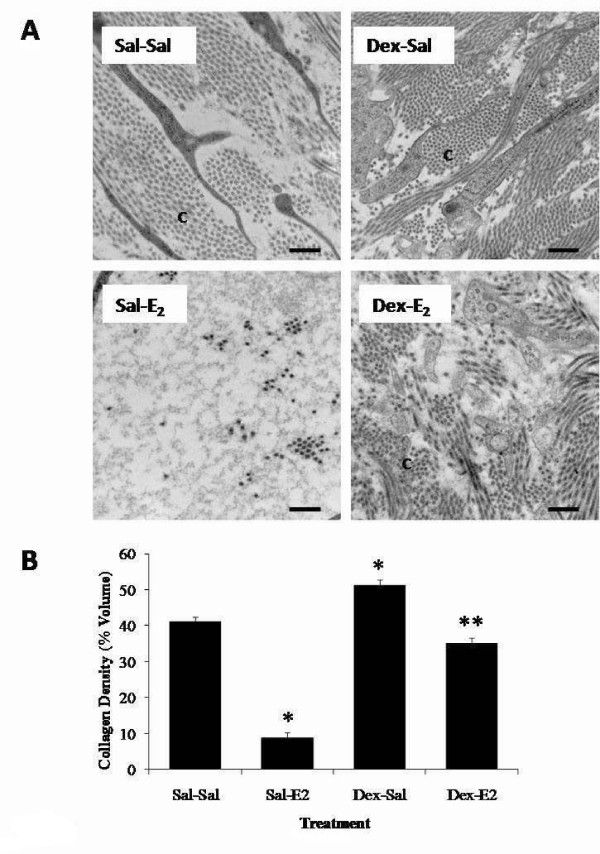
**Effects of dexamethasone pretreatment on E_2_-induced changes in uterine stromal matrix density**. Transmission electron microscopy analysis of endometrial stroma from animals pretreated with saline or dexamethasone (Dex: 600 μg/kg) 20 h and 4 h prior to administration of E_2 _or saline injected 4 h prior to tissue collection. Panel A: Representative TEM micrographs of stromal matrix. Micrographs shown are typical from a total of 54 to 70 images analyzed from 3-4 animals per treatment group; C=Collagen, scale bar = 0.5 μm. Panel B: Quantified endometrial collagen density values representing mean percent volume for n = 3-4 animals per treatment group, total micrographs analyzed per group range from 54 to 70; one-way ANOVA, error bars represent ± SE; *P < 0.05 compared to saline control (Sal-Sal), **P < 0.05 compared to E_2 _control (Sal-E_2_).

Because peak changes in matrix density and cytokine expression correlate temporally with the hypertrophic phase of E_2_-induced uterine growth, it was of interest to assess the effects of the anti-inflammatory synthetic glucocorticoid, dexamethasone (600 μg/kg) on this inflammatory-like response. Dexamethasone pretreatment alone induces a decrease in uterine wet weight and significantly inhibits the typical increase in uterine wet weight effected by E_2 _(Figure 7) in a manner consistent with previously published reports [34]. With respect to stromal matrix density, dexamethasone alone produces both a qualitative and quantitative change in matrix structure (Figure 8); there is a noticeable and statistically significant increase in stromal matrix density as compared to saline control animals (Dex-Sal vs. Sal-Sal). This indicates that dexamethasone modulates stromal matrix remodeling so as to generate a more densely organized collagen architecture in the immature uterus. In addition, dexamethasone pretreatment inhibits E_2_-induced matrix remodeling; stromal collagen density is much higher (~4-fold) in dexamethasone-pretreated (Dex-E_2_) animals as compared to saline-pretreated E_2_-challenged animals (Sal-E_2_). Quantitative analysis of collagen density indicates that collagen levels are equivalent in the Dex-E_2 _as compared to control animals (Sal-Sal). It should be noted that the magnitude of inhibition of collagen matrix remodeling induced by E_2 _is smaller in dexamethasone pretreated animals (Figure 8 bottom, Dex-E_2 _vs. Sal-E_2_) indicating that it is not fully efficacious at inhibiting the E_2 _response.

### Dexamethasone pretreatment modifies E_2_-induced changes in uterine MMP expression

**Figure 9:**
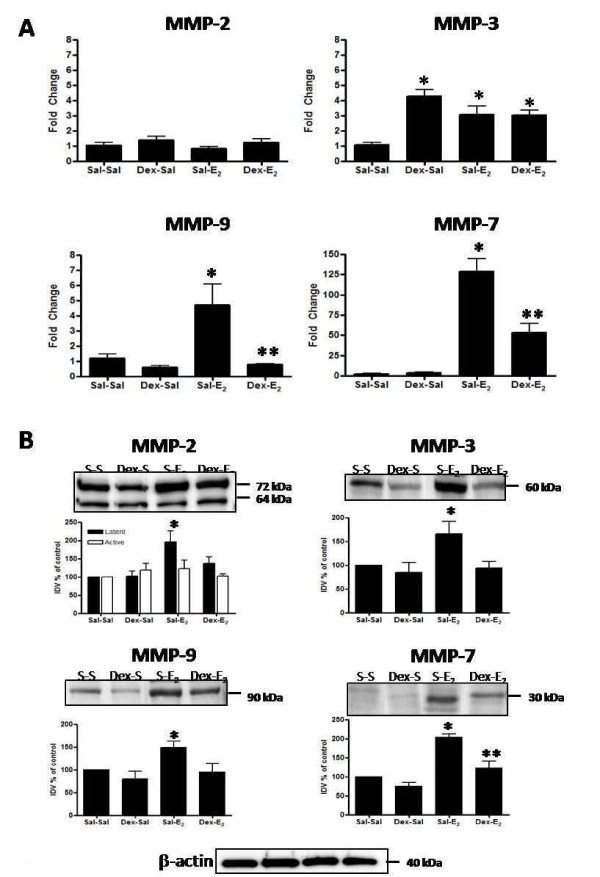
**Dexamethasone effects on E_2_-induced changes in MMP mRNA and protein**. Uterine mRNA analysis via TaqMan^® ^real-time PCR (panel A) and protein analysis via Western blot (panel B) are shown for MMP-2, MMP-3, MMP-7 and MMP-9 from animals pretreated with saline or dexamethasone (Dex: 600 μg/kg) 20 h and 4 h prior to administration of E_2_(40 μg/kg) or saline given 4 h before tissue collection. Messenger RNA values (panel A) represent mean ± SE, n = 5-6 per group; one way ANOVA, *P < 0.05 compared to saline control (Sal-Sal), **P < 0.05 compared to E_2 _control (Sal-E_2_). Blot images shown (panel B) are representative of a minimum of 3 blots using 3 separate pools of total protein extracted from different animals. Intensities of protein bands were quantitated using AlphaEase FC software (Alpha Innotech) as described in the Methods and values for each time point expressed as percent of saline control (100%). Quantitative data (panel B) reflect average band intensity values ± SE measured from ≥ 3 blots with total protein extracted from different animals in 2 separate experiments; one way ANOVA, *P < 0.05 compared to saline control (Sal-Sal), **P < 0.05 compared to E_2 _control (Sal-E_2_). IDV = integrated density value. Western blot analysis of β-actin as a control for gel loading is shown for the protein samples used to assess MMP protein for the blots above.

In light of the inhibitory effect on E_2_-induced stromal matrix remodeling (Figure 8), MMP mRNA and protein levels were analyzed in animals pre-treated with dexamethasone. Dexamethasone treatment increases expression of MMP-3 mRNA, when compared to levels in control animals (Figure 9A; Dex-Sal vs. Sal-Sal). In addition, dexamethasone does not inhibit the E_2_-induced increase in MMP-3 mRNA (Figure 9A; Dex-E_2 _vs. Sal-E_2_). Levels of mRNA in Dex-E_2 _animals are not significantly different from those in Dex-Sal controls, indicating that dexamethasone induction of mRNA expression for MMP-3 is not modified in the presence of E_2_. Transcript levels for MMP-7 and MMP-9 are unaffected by pretreatment (Figure 9A, Dex-Sal vs. Sal-Sal), although dexamethasone pretreatment does significantly inhibit the E_2_-induced upregulation of mRNA for these two enzymes (Dex-E_2 _vs. Sal-E_2_). Dexamethasone does not induce a change in MMP-2 mRNA (Figure 9A); however, effects on the E_2_-induced upregulation of MMP-2 mRNA were not assessed since only the 4 h post-hormone treatment time point was studied, and transcript levels for this enzyme do not significantly increase until 48 h post-E_2 _administration (Figure 4A).

**Figure 10:**
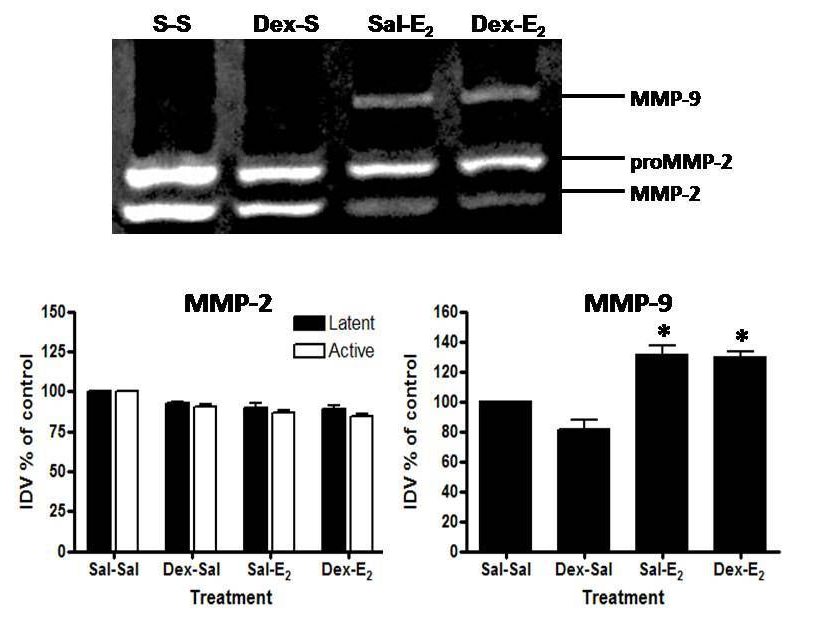
**Effect of dexamethasone on E_2_-induced changes in gelatinase activity**. Gelatin zymography analysis is shown for MMP-2 and MMP-9 activity from animals pretreated with saline or dexamethasone (Dex: 600 μg/kg) 20 h and 4 h prior to administration of E_2 _(40 μg/kg) or saline given 4 h before tissue collection. Zymogram (top) is representative of 6 gels using 3 separate pools of total protein extracted from different animals. Quantitative data (bottom) represents analysis of band intensity using AlphaEase FC software (Alpha Innotech) as described in the Methods; one way ANOVA, values represent mean ± SE for 6 gels. *P < 0.05 compared to saline control (Sal-Sal).

Western blot analyses of MMP protein levels support the effects of dexamethasone on mRNA expression. Pretreatment inhibits E_2_-induced upregulation of protein expression for MMP-3, MMP-7, and MMP-9 (Figure 9B; Dex-E_2 _vs. Sal-E_2_). Interestingly, levels of MMP-3 protein do not increase in response to dexamethasone pretreatment despite evidence of an elevation of tissue mRNA (Figure 9A). Gelatin zymography shows that dexamethasone pretreatment did not affect activity of MMP-2 or MMP-9 (Figure 10), although dexamethasone pretreatment does decrease the E_2_-induced upregulation of MMP-9 total protein levels (Figure 9B). Because full activity of MMPs may not be recovered as a result of refolding of the enzymes after electrophoresis in gel zymography [51], it is unclear whether dexamethasone affects levels of active MMP-9 in vivo.

### Dexamethasone pretreatment affects E_2_-induction of uterine MCP-1 and TNF-α expression

**Figure 11:**
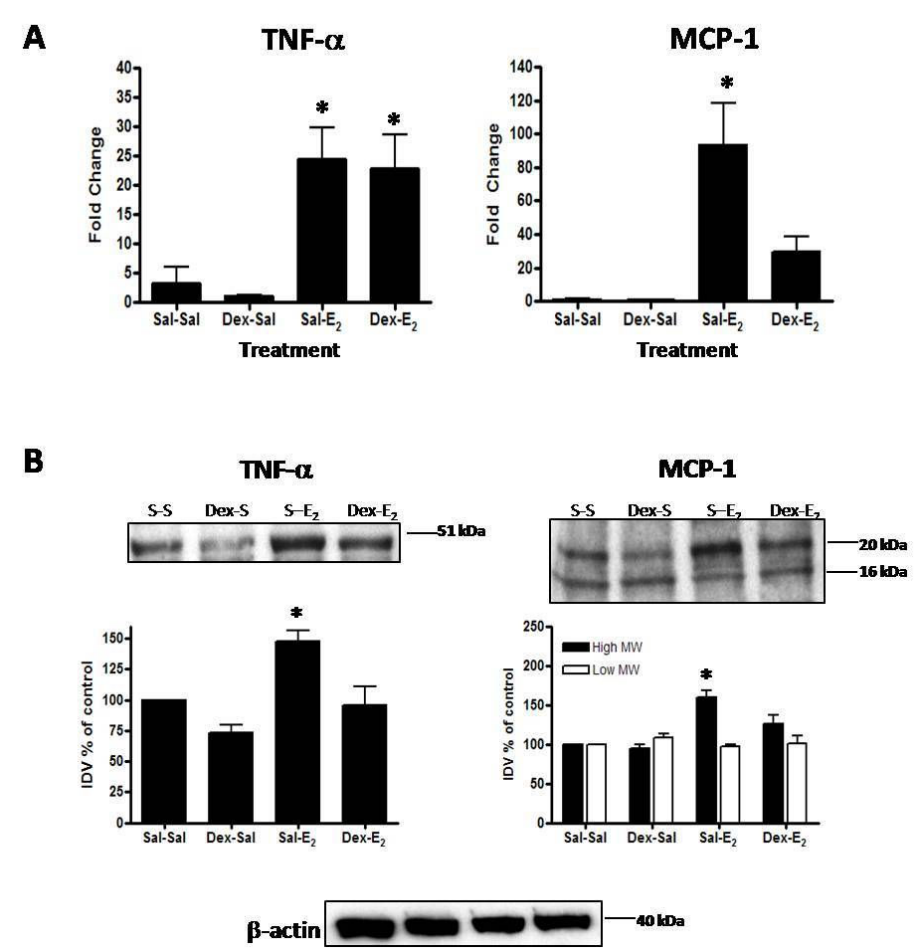
**Dexamethasone effects on E_2_-induced changes in cytokine mRNA and protein**. Uterine mRNA analysis via TaqMan^® ^real-time PCR (panel A) and protein analysis via Western blot (panel B) are shown for TNF-α and MCP-1 from animals pretreated with saline or dexamethasone (Dex: 600 μg/kg) 20 h and 4 h prior to administration of E_2_(40 μg/kg) or saline given 4 h before tissue collection. Messenger RNA values (panel A) represent mean ± SE, n = 5-6 per group; one way ANOVA, *P < 0.05 compared to saline control (Sal-Sal). Western blot images shown (panel B) are representative of a minimum of 3 blots using 3 separate pools of total protein extracted from different animals in 2 separate experiments. Intensities of protein bands were quantitated using AlphaEase FC software (Alpha Innotech) as described in the Methods and values for each time point expressed as percent of saline control (100%). Quantitative data (panel B) reflect average band intensity values ± SE measured from ≥ 3 blots with total protein extracted from different animals in 2 separate experiments; one way ANOVA, *P < 0.05 compared to saline control (Sal-Sal), **P < 0.05 compared to E_2 _control (Sal-E_2_). IDV = integrated density value. Western blot analysis of β-actin as a control for gel loading is shown for the protein samples used to assess TNF-α and MCP-1 protein for the blots above.

Dexamethasone potently inhibits the E_2_-induced upregulation of MCP-1 mRNA and protein observed at 4 h post-hormone treatment (Figure 11). However, pretreatment does not affect E_2_-induced upregulation of TNF-α mRNA (Figure 11A), though it does decrease protein content (Figure 11B).

## Discussion

An examination of tissue responses in the E_2_-treated OVX rat provides an opportunity to study selective effects of the hormone that can be defined in the temporal context of the hypertrophic and hyperplastic growth responses. In this model, E_2 _induces uterine growth that is characterized by changes in endometrial tissue architecture and gene expression. We used TEM analysis to more specifically assess changes in stromal collagen matrix quantitatively. A significant decrease in matrix density is observed as early as 45 minutes and reaches its lowest value, with an approximate 70% reduction of the original collagen network, at 4 hours post-hormone treatment, coinciding with the peak time point of uterine hypertrophy. This indicates that activation of matrix-processing enzymes is extremely rapid and implies regulation of inherent proteases through an estrogen receptor (ER)-mediated pathway.

Pretreatment with the broad-range MMP inhibitor batimastat effectively ablates E_2_-induced changes in stromal ultrastructure supporting a critical role for these enzymes in tissue remodeling. These results differ from those by Kaitu'u and colleagues [38], who reported that batimastat reduced MMP activity but did not alter endometrial breakdown or repair as assessed through light microscopy in a mouse decidualization model. The contrasting results may reflect a difference in matrix processing during E_2_-specific induction of tissue growth in the immature uterus as compared to tissue regression following progesterone withdrawal in a decidualization model. In addition, analysis of fine matrix structure and density using quantitative TEM provides a more sensitive measure of changes in collagen organization indicative of tissue remodeling as compared to qualitative assessment of tissue appearance via light microscopy [46].

While a number of MMPs are known to be expressed in cell-type as well as cycle-specific patterns in the uterus [2,5,50], E_2_-specific regulation in an OVX rodent model has been studied in vivo for only a few [19]. Using this model we show that expression of MMP-3, MMP-7, and MMP-9 is upregulated at both RNA and protein level early within the E_2_-induced growth response correlating with the time point where stromal collagen matrix density reaches its lowest point (4 h). While substrates for these enzymes include fibrillar, fibril-associated, and network forming collagens [50], in vivo substrates are unknown. Therefore, although expression correlates with maximum matrix remodeling temporally, we cannot conclude that these particular proteases are directly causative. As an epithelial cell expressed protease, MMP-7 is likely not critically involved in stromal matrix processing directly, whereas MMP-3 and MMP-9 are known to be expressed by stromal cells or infiltrating leukocytes [50]. Expression of MMP-2 increases most significantly later in the growth response (48 h) suggesting a complex level of regulation that may involve E_2_-triggered production of signal compounds which, in turn, enhance MMP-2 production. Interestingly, tissue levels of MMP-3 protein also remain elevated late in the growth response (24-72 h), indicating extended in-tissue stabilization of this enzyme. The overall elevation of MMP-2 and MMP-3 content late, as compared to MMP-7 and MMP-9 protein which peak early in the growth response, implies alternate temporal functions for tissue MMPs that may include activation of matrix processing during both tissue growth and regression phases. While providing important insight into the E_2_-triggered response at the whole tissue level, our analysis of changes in MMP mRNA and protein content does not define stromal-specific changes in enzyme content and activity. Future studies to analyze MMP activity via in situ zymography and mRNA expression using laser capture microdissection and subsequent gene profiling would provide a more precise window into the stromal tissue response.

While we observed transient upregulation of MMP-2 and MMP-9 mRNA and protein expression in response to E_2 _in the OVX immature rat uterus, Zhang and colleagues [19] did not observe an effect of E_2 _on MMP-2 mRNA levels in the OVX adult mouse through 24 h post-hormone treatment, though they did observe a decrease in MMP-9 mRNA, but not protein activity, 4-8 h post-treatment. Since we observe peak changes in MMP-2 mRNA expression at 48 h after E_2 _administration, it is unclear if a similar response would be evident in an OVX adult model as that time point was not assessed [19]. In contrast, our analysis of MMP-9 mRNA protein via both Western blot and zymography clearly shows upregulation by 4 h post-E_2 _administration. Data inconsistencies may reflect differences in rodent model (mouse vs. rat) or in tissue responsiveness between the immature (first time hormone exposure) as opposed to an adult (hormone-primed) uterus.

A variety of inflammatory mediators [8,9,40,52,53] have been shown to regulate expression and activity of MMPs, and, E_2 _is known to induce an acute inflammatory-like response within the uterus [54]. We show that E_2 _induces potent and rapid upregulation of TNF-α, with peak increases in mRNA and protein occurring at the time point where MMP expression and stromal matrix processing are upregulated (4 h). It is difficult to understand the exact role of TNF-α expression in E_2_-induced uterine matrix remodeling or in MMP gene transcription as this bioactive compound is expressed in tissues and held in an inactive state through cell surface or extracellular matrix-binding interactions. Although ADAM-17 (TACE) is the best characterized TNF-α sheddase, MMP-2, MMP-3, MMP-7, and MMP-9 have been shown to release TNF-α from the cell surface in a similar mechanism [13,45]. Protease-mediated shedding of TNF-α may induce signal pathway activation that precedes an overall upregulation of mRNA and protein expression for this inflammatory mediator, thus analysis of expression alone is not a full indicator of TNF-α activity in vivo. A study of E_2_-induced changes in activity of the TNF-α pathway, including activation of NF-κB, will be important for a better understanding of the role of this inflammatory agent as a critical regulator of MMP expression in the estrogen response pathway.

Expression of the β-chemokine MCP-1 (CCL2), a potent chemoattractant for macrophages, T cells, basophils, and mast cells is also potently upregulated at 4 h. Enhanced production of MCP-1 is known to occur during the proliferative phase of the cycling uterus and, expression is stimulated by TNF-α [55-57]. The timing of highest tissue levels of MCP-1 at 4 h correlates with the peak in expression of TNF-α, suggesting that it may be a key signal component for inducing leukocyte influx by creating in-tissue gradients of chemokines, like MCP-1, which are triggered early during the hypertrophic phase of uterine growth. MCP-1 is also a substrate for a variety of MMPs, including MMP-3 [14], and protease-mediated processing has been shown to inhibit bioactivity of β-chemokines, thereby regulating the timing and intensity of leukocyte influx once an inflammatory response is underway [14,44,45,58].

Correlations between E_2_-regulated uterine growth, inflammation, and MMP expression and activation in the immature OVX uterus led us to investigate the effects of dexamethasone, the synthetic glucocorticoid and anti-inflammatory agent. We show that dexamethasone modifies stromal matrix collagen density and, pretreatment with dexamethasone effectively inhibits E_2_-induced collagen remodeling. These data suggest that the regulation of collagen density is under the influence of activated glucocorticoid-receptor (GR) and, implicates the MMP system because dexamethasone is known to inhibit MMP expression [59]. Dexamethasone effects on MMP expression however, are complex. Of the MMPs that are upregulated 4 h post-E_2 _treatment, dexamethasone pretreatment inhibits both MMP-7 and MMP-9 mRNA and protein expression but does not inhibit the E_2_-induced increase in MMP-3 mRNA or protein.

In addition to effects on the MMP system, dexamethasone is also known to block the pro-inflammatory effects of E_2 _in the uterus [34]. Glucocorticoids have been shown to suppress expression of chemoattractant chemokines, such as MCP-1 [60], and possibly, through inhibition of MMP expression, may decrease sheddase activity and ECM processing important for liberation of inherent bioactive agents, including TNF-α, that activate signal pathways to enhance inflammatory processes. Our data clearly show that dexamethasone has no effect on TNF-α or MCP-1 mRNA or protein expression. However, dexamethasone pretreatment potently inhibits E_2_-induced upregulation of MCP-1 mRNA and protein expression and decreases levels of TNF-α protein observed at 4 h post-treatment, thereby supporting its effects as a suppressor of both inflammatory signaling as well as matrix remodeling.

Collectively our data show that E_2_-induced endometrial matrix remodeling, production of MMPs, and inflammatory mediators correlate temporally with the early phase of uterine growth which is hypertrophic and inflammatory-like in nature. Dexamethasone as an anti-inflammatory agent inhibits these processes however, because activated GR can directly modify expression of MMPs and inflammatory agents such as MCP-1, further investigation is necessary to determine the role of inflammation itself on matrix remodeling. One model of the E_2 _response would be that hormone-triggered rapid activation of inherent proteases leads to matrix processing that liberates bound inflammatory mediators like TNF-α, thereby producing inflammation and leukocyte influx. Alternatively, rapid E_2_-induced inflammation triggered by ER signal pathways and production of paracrine regulators like prostaglandins would drive influx of leukocytes which, in turn, activate inherent proteases to effect changes in stromal matrix architecture necessary for tissue expansion as growth proceeds. Evidence indicates that infiltrating leukocytes contribute to tissue protease density [8,9,61-63] and neutrophilic depletion clearly inhibits endometrial repair in the mouse [25]. Further studies must focus upon the timing of E_2_-induced changes in expression and activity of inflammatory mediators and leukocyte influx in relation to matrix processing to more clearly delineate the E_2 _response pathway. In addition, the use of other kinds of anti-inflammatory agents such as COX inhibitors, that would specifically inhibit production of prostaglandins, will also provide a better understanding of the role of inflammatory reactions per se on matrix processing.

## Conclusion

This study using the OVX immature rat model contributes to a better understanding of specific regulation of MMP expression and endometrial tissue remodeling induced by E_2 _and the counter-regulatory effects of dexamethasone. We have established a novel assay system for measuring stromal collagen matrix density as a reliable and specific endpoint for assessment of matrix remodeling. The data demonstrate that E_2_-regulated endometrial remodeling is rapid in onset (<1 hour) and peak changes in expression of MMP-3, -7, and -9 and the inflammatory mediators TNF-α and MCP-1, correlate temporally with the period of lowest stromal collagen density during uterine tissue hypertrophy (4 h). Our survey of changes in expression of MMPs and inflammatory mediators provides the groundwork for further studies of molecular triggers that link ER activation with inflammatory-like responses and matrix processing in the uterus.

## Competing interests

The authors declare that they have no competing interests.

## Authors' contributions

LAR participated in the design of the study, in directing and completing all experimental analyses and in writing the manuscript. BJP participated in MMP mRNA analyses, in TEM tissue preparations and in writing the manuscript. SPT contributed significantly to generation of TEM data, mRNA analyses, and figure preparations. TDC developed the TEM quantitative method and participated in cytokine analyses to assess temporal changes in response to E_2_. BAO completed initial analyses of MMP expression and batimastat inhibitor studies. JAC contributed to quantitative TEM analysis for dexamethasone studies. ARS and OV participated in animal treatments and tissue preparations for MMP expression analysis. RMG participated in planning experiments and contributed to drafting of the manuscript. All authors have read and approved the final manuscript.
